# Synergistic antibacterial action of the iron complex and ampicillin against *Staphylococcus aureus*

**DOI:** 10.1186/s12866-023-03034-1

**Published:** 2023-10-06

**Authors:** Ludmila Kosaristanova, Martin Rihacek, Frantiska Sucha, Vedran Milosavljevic, Pavel Svec, Jana Dorazilova, Lucy Vojtova, Peter Antal, Pavel Kopel, Zdenek Patocka, Vojtech Adam, Ludek Zurek, Kristyna Dolezelikova

**Affiliations:** 1 Department of Chemistry and Biochemistry, Faculty of AgriSciences, Mendel University in Brno, Brno, Czech Republic; 2 Central European Institute of Technology, University of Technology, Brno, Czech Republic; 3 Department of Inorganic Chemistry, Faculty of Science, Palacky University, Olomouc, Czech Republic; 4 Department of Forest Management and Applied Geoinformatics, Faculty of Forestry and Wood Technology, Mendel University in Brno, Brno, Czech Republic

**Keywords:** *Staphylococcus aureus*, Iron complex, Ampicillin, Synergy, Antimicrobial activity

## Abstract

**Objectives:**

Resistance to antibiotics among bacteria of clinical importance, including *Staphylococcus aureus,* is a serious problem worldwide and the search for alternatives is needed. Some metal complexes have antibacterial properties and when combined with antibiotics, they may increase bacterial sensitivity to antimicrobials. In this study, we synthesized the iron complex and tested it in combination with ampicillin (Fe16 + AMP) against *S. aureus*.

**Methods:**

An iron complex (Fe16) was synthesized and characterized using spectroscopy methods. Confirmation of the synergistic effect between the iron complex (Fe16) and ampicillin (AMP) was performed using ζ*–*potential, infrared spectra and FICI index calculated from the minimum inhibitory concentration (MIC) from the checkerboard assay. Cytotoxic properties of combination Fe16 + AMP was evaluated on eukaryotic cell line. Impact of combination Fe16 + AMP on chosen genes of *S. aureus* were performed by Quantitative Real-Time PCR*.*

**Results:**

The MIC of Fe16 + AMP was significantly lower than that of AMP and Fe16 alone. Furthermore, the infrared spectroscopy revealed the change in the ζ*–*potential of Fe16 + AMP. We demonstrated the ability of Fe16 + AMP to disrupt the bacterial membrane of *S. aureus* and that likely allowed for better absorption of AMP. In addition, the change in gene expression of bacterial efflux pumps at the sub-inhibitory concentration of AMP suggests an insufficient import of iron into the bacterial cell. At the same time, Fe16 + AMP did not have any cytotoxic effects on keratinocytes.

**Conclusions:**

Combined Fe16 + AMP therapy demonstrated significant synergistic and antimicrobial effects against *S. aureus*. This study supports the potential of combination therapy and further research.

**Supplementary Information:**

The online version contains supplementary material available at 10.1186/s12866-023-03034-1.

## Introduction

The emergence of antimicrobial resistance is a problem causing a global crisis and the rate of development of new antimicrobial agents is not adequate. Consequently, infections caused by multidrug-resistant bacteria including *Staphylococcus aureus* are of a great concern worldwide [1]. Staphylococcal infections in humans and animals are commonly treated with β-lactam antibiotics, including ampicillin (AMP) [2]. A great selective pressure from the intensive and extensive use of antibiotics has resulted in β-lactam resistance based on several mechanisms such as efflux pumps, siderophores, and production of β-lactamases [2–4]. Currently, the majority of clinical strains of *S. aureus* are β-lactamase positive [2], use efflux pumps, and also produces siderophores [3, 4].

Antimicrobial properties of metals have been known for several decades [5] and their combination with antibiotics has been assessed in several studies. For example, it was shown that β-lactams together with silver or zinc oxide nanoparticles had great antimicrobial activity against various multidrug resistant bacteria [6, 7]. Iron has been also considered as a potential candidate for combined treatment with antibiotics [8]. Iron is the most abundant transition element in the human body and a promising antimicrobial agent in the form of a metal complex. Iron in the form of a metal complex can affect bacterial cells where they cause oxidative stress, inhibit respiratory processes and ATP production, increase cell hydrophobicity, and facilitate their penetration across the cell wall [7–9]. Therefore, combined treatments offer several advantages such as a lower potential for developing resistance, additive or synergistic effects, increasing the effectivity of antibiotics, and overcoming drug resistance [10]. Combination therapy of an iron complex with β-lactams has been tested previously against *Escherichia coli* and it was more effective than antibiotics alone [9].

The goal of this study was to synthesize the iron complex Fe16 and test it in combination with AMP against *S. aureus*. We also aimed to investigate the mechanism of the synergistic action of Fe16 + AMP against *S. aureus* and to evaluate whether it has any negative effects on eukaryotic cells represented by the HaCaT cell line.

## Results

### Physico-chemical characterization of Fe16 complex and interaction between Fe16 and AMP

The infrared spectrum of Fe16 exhibited characteristic bands of the Fe(II)-tris(diimine) complexes. Also, the electronic spectra of Fe16 showed absorption bands characteristic for Fe(II)-tris(diimine) complexes (Fig. S1Aa,b and Table S2). Interactions between Fe16 and AMP were confirmed by the ζ–potential and ATR-FTIR. The ζ–potential of Fe16 alone was + 55.8 mV and AMP alone was -16.5 mV. The ζ–potential of Fe16 + AMP was shifted toward slightly negative values -4.2 mV (Fig. S2) indicating the formation of new complexes. To further study the interactions between Fe16 and AMP in the Fe16 + AMP complex, spectra of Fe16, AMP, and Fe16 + AMP were recorded by ATR-FTIR (Fig. S1C). No major spectral alterations were observed comparing the Fe16 spectrum and the Fe16 subtracted spectra. With the focus on AMP and AMP subtracted spectra, the two significant band broadenings corresponding to valence carboxylate vibrations (COO^−^) at the wave number values of 1 590 and 1 370 cm^−1^ were observed in spectrum of Fe16 + AMP. These carboxylate groups mediate the interaction in Fe16 + AMP complex.

### An antibacterial efficacy and synergistic effect of Fe16, AMP, and Fe16 + AMP on *S. aureus* by the checkerboard assay

**Fig. 1:**
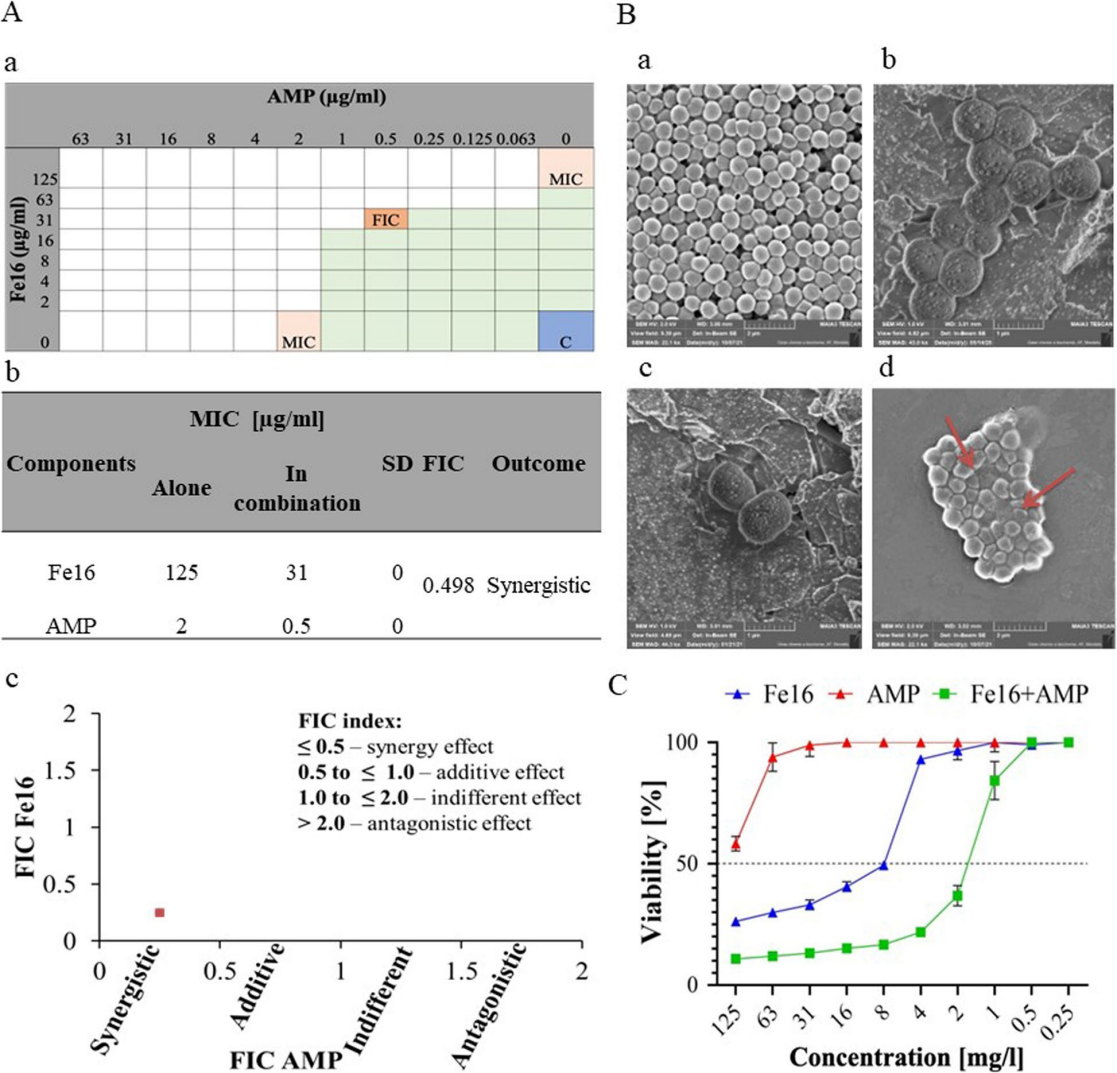
**Aa** Visualization of the checkerboard format: Green boxes represent growth and white boxes inhibition. Orange box shows Fractional inhibition index, pink boxes demonstrate positions of minimal inhibitory concentrations of Fe16 and AMP. Blue box is no-treated *S. aureus* (Control). **Ab** image presents antibacterial and synergy activity with MIC and FIC results from checkerboard assay. The values are presented as the average from three independent experiments. **Ac** Isobologram showing the synergy effect of Fe16 and AMP with FIC index ≤ 0.5 and table demonstrating the the FIC index category scale. **B** External morphological changes of *S. aureus* after exposure to Fe16+AMP assessed by SEM. Figure **Ba** shows untreated *S. aureus* as a control, **Bb** demonstrates *S. aureus* treated with Fe16 and Bc AMP alone at 0.125 ug/ml. **Bd** is *S. aureus* treated with Fe16+AMP at 0.25 ug/ml. Red arrows indicate *S. aureus* morphological changes. **C** Cytotoxic effects of Fe16, AMP and Fe16+AMP at the different concentrations on the HaCaT keratinocyte cell line

The inhibitory effect from the checkerboard assay of Fe16 + AMP against *S. aureus* was significantly greater than that of the individual compounds (Fig. 1A). The MIC value of Fe16 31 µg/ml and AMP 0.5 µg/ml in combination against *S. aureus* was significantly lower than that of Fe16 and AMP alone with MIC values of 125 µg/ml and 2 µg/ml (Fig. 1Ab). This finding also correlated with the results of the FIC index. Isobologram showed the synergistic effect between Fe16 (FIC_Fe16_ = 0.248) and AMP (FIC_AMP_ = 0.250) (Fig. 1Ac) based on the FIC index calculation of the two tested components. The antibacterial effect of the Fe16 + AMP and its synergistic effect (≤ 0.5) against *S. aureus* was confirmed by the FIC index 0.498.

### Observation of morphological changes of *S. aureus* after Fe16, AMP and Fe16 + AMP treatment

SEM microscopy of *S. aureus* treated with Fe16 + AMP revealed that the integrity of the cell wall was compromised. In contrast, untreated *S. aureus* cells retained their coccus morphology and the cell surface was compact (Fig. 1Ba). No morphological change was also observed in *S. aureus* cells treated with Fe16 (Fig. 1Bb) and AMP alone (Fig. 1Bc). On the other hand, significant morphological changes were observed after 24 h on *S. aureus* cells treated with Fe16 + AMP at the sub-inhibitory concentration (0.25 µg/ml) showing damaged cells with disrupted walls and membranes (Fig. 1Bd).

### Cytotoxicity properties of Fe16, AMP and Fe16 + AMP

The cytotoxic effect of Fe16 on the HaCaT keratinocyte cell line was observed in the concentration range of 8–125 µg/ml, whereas for AMP no cytotoxicity was observed (Fig. 1C). At the concentration of 0.5 µg/ml of Fe16 + AMP the viability of keratinocytes was 90.0% (Fig. 1C).

### Changes in the regulation of selected genes of efflux pumps, β-lactamase and ABC transporters after treatment with Fe16, AMP and Fe16 + AMP

**Fig. 2:**
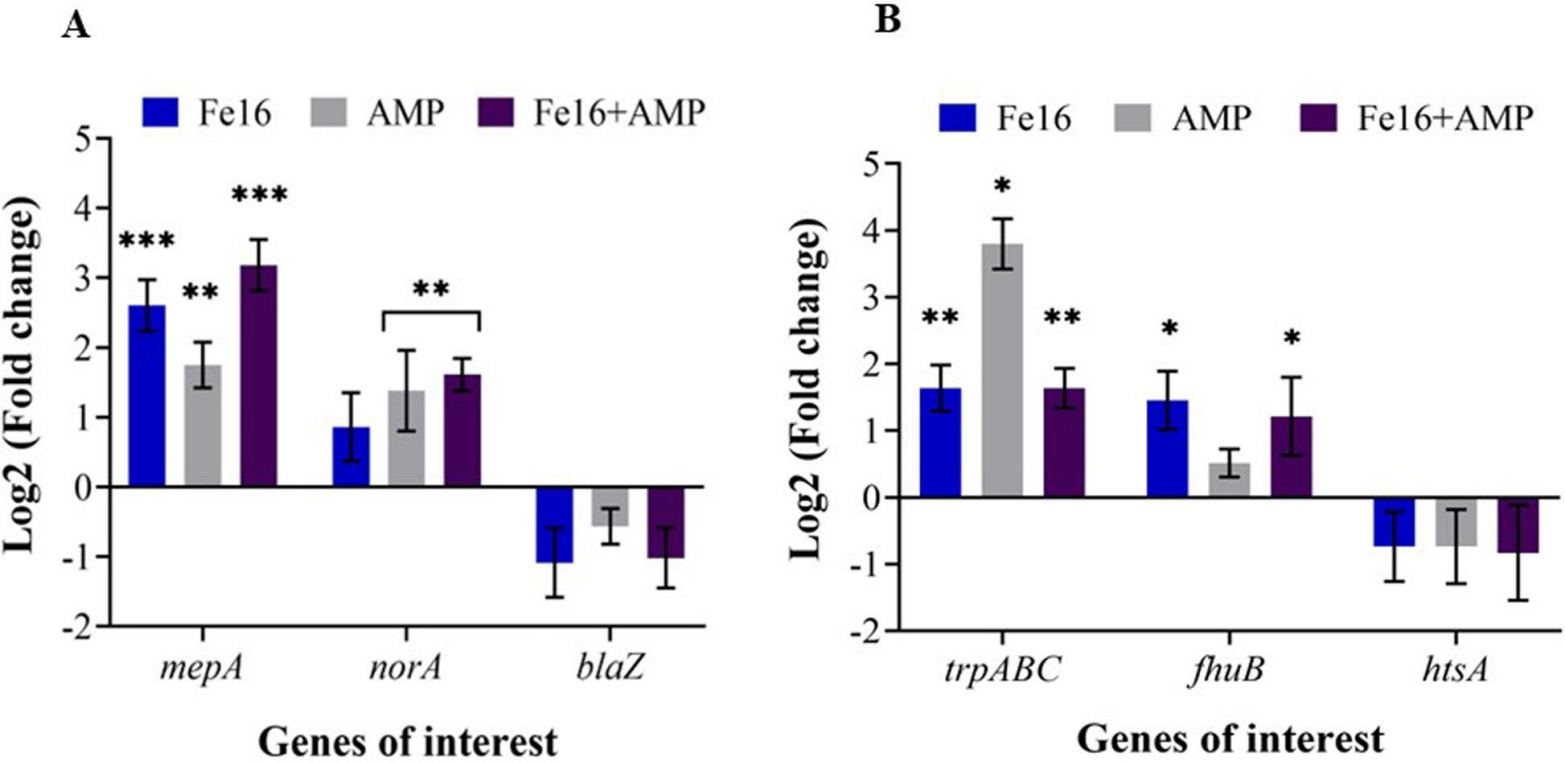
Gene expression a Log2 (fold change) of the ABC transporter family (**A**) and defense system of *S. aureus* after exposure to AMP, Fe16 and Fe16 + AMP (**B**). Significant changes of Fe16, AMP and Fe16 + AMP in comparison to that of control are marked with asterisks: *p-value < 0.05, **p-value < 0.01, ***p-value < 0.001

The expression of the efflux pump gene *mepA* significantly increased after all three treatments (fold change in the range 3.43—9.33) although the effect of AMP alone was at significance level *p*-value < 0.01. The expression of the *norA* gene also significantly increased after AMP (2.8 fold change) and Fe16 + AMP (3.1 fold change) treatments (Fig. 2A). Gene expression of *blaZ* was downregulated, although not significantly (*p*-value > 0.2) (Fig. 2A). The significantly increased expression was detected for the transporter genes *trpABC* and *fhuB* after exposure to Fe16 and Fe16 + AMP (*p*-value < 0.05) (Fig. 2B). Ampicillin alone affected the expression of *trpABC* only. The expression of the third ABC transporter gene, *htsA*, was downregulated non-significantly after all treatments (Table S3).

## Discussion

In recent decades, many metal-based complexes have been tested for their antimicrobial properties [1, 8]. It has been shown that iron exhibits antimicrobial effects against Gram-positive and Gram-negative bacteria in the form of metal complex or nanoparticles [8, 11].

In the synthetized complexes structure [12], the difference in wavelengths observed for asymmetric and symmetric ν(COO^−^) vibrations indicates that the compound Fe16 adopt ionic structure and fumarate ions are not coordinated to the central atoms. The strong bands can be assigned to asymmetric *ν*(COO^−^) vibrations but suffer interference with C = C and C = N stretching vibrations of aromatic ring [13]. Considering that pK_a_ values of AMP functional groups (2.5/7.3) the carboxylic acid became anionic (COO¯), and amine group cationic (NH_34_^+^) [14]. However, with increase the pH over the AMP pK_a_ values the targeted Fe16 complex after interaction with AMP became deprotonated (anionic form), which explains the strong decrease of the Fe16 complex positive charge and slight increase of negative surface charge of newly formed complexes. Due to the shift of carboxylate asymmetric valence vibrations to higher wavenumbers and symmetric valence vibration to lower numbers are observed in FTIR analysis. These spectral variations are connected to the carboxylate group passing from the free zwitterionic COO^−^ group to organometallic coordination in the Fe16 + AMP [15]. The band shifts in spectral region suggest interaction of AMP phenyl with nphen in Fe16 via π–π electron donor–acceptor complex system [16], while the vibration positions related to β-lactam ring in AMP as well as NO_2_ functional group in Fe16 remains unchanged. These data indicate that the key antibacterial structure in the complex remains unaffected [17].

The antimicrobial effect of Fe16 + AMP on *S. aureus* showed synergistic activity at thre phenotypic and transcriptomic level. Previously, it was shown that other metals such as copper, zinc, and iron in combination with amoxicillin increased effectiveness against *E. coli* [9]. Although susceptibility of bacteria to doped antibiotics with metal complexes has been shown before [18], investigations on the iron complex are rare [8]. Cell integrity of *S. aureus* treated with Fe16 + AMP was compromised leading to cell death which corroborates similar study with silver nanoparticles and antibiotics [19]. Metal complexes increase the lipophilic character of the transition metal ion and therefore increase the hydrophobicity and facilitate their penetration through the bacterial cell wall and membrane [9]. In our case, Fe16 + AMP appears to facilitate the penetration of AMP through the cell wall of *S. aureus*. Study of Panacek et al. (2016) showed that with AgNps silver nanoparticles in combination with antibiotics including β-lactams had also synergistic effect against *S. aureus* [20].

It is important to note that no cytotoxic effects against the HaCaT cell line after exposure to Fe16 + AMP was observed. In recent years, many neurological disorders have been attributed to iron overload; however, further studies have optimized new complex metal compounds in combination with various materials to moderate general cytotoxicity [21, 22].

**Fig. 3:**
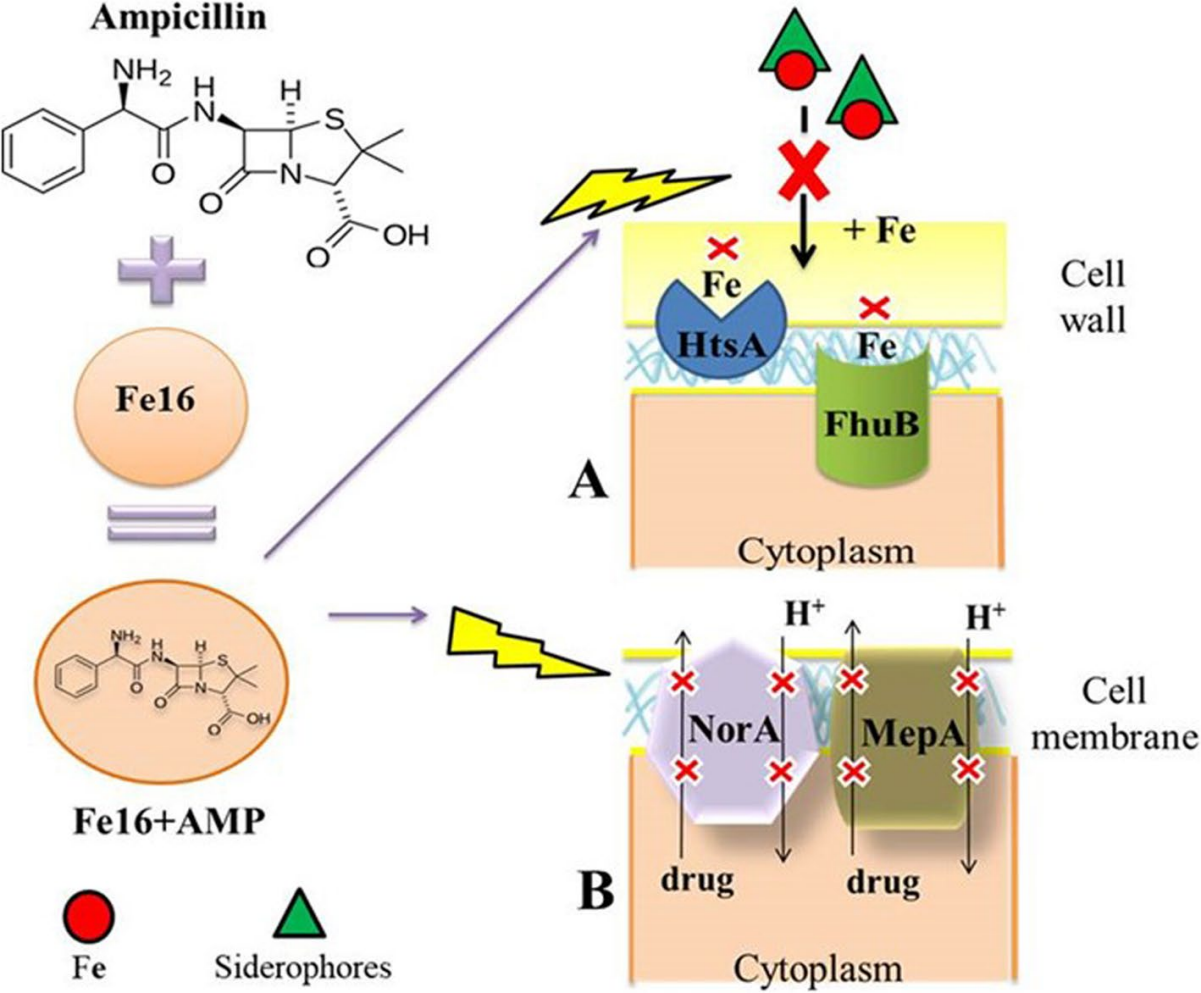
Mechanism of the antimicrobial effect of Fe16 + AMP causing stress in *S. aureus* and disruption of function of efflux pumps and ABC transporters **A**. Import of iron into cells, needed by *S. aureus* for growth and metabolism, is disrupted. **B** NorA and MepA efflux pumps are not sufficient to push Fe16 + AMP out of cells

The combination of Fe16 + AMP at the sub-inhibitory concentration also likely led to disruption of the efflux pumps and that is consistent with the MICs results. The study of the inhibitory effect of efflux pumps under the influence of the iron complex alone and in combination with antibiotics has not been conducted previously. However, binding of iron oxide nanoparticles with rifampicin to the active site of the efflux pump and consequent blocking of its function has been demonstrated [23]. Termination of the proton gradient, followed by disruption of the membrane potential and/or loss of proton motive force can lead to failure of the driving force, which is essential for the function of efflux pumps [24, 25]. It is also assumed that iron nanoparticles disrupt the activity of efflux pumps by generating ROS [25]. Large metal oxides have the ability to induce faster electron transfer kinetics to the active site of enzymes [26]. Similarly, upregulation of *mepA* has been reported using silver nanoparticles and subsequent exposure to Ag^+^ ions [27]. Although the highest expression of *mepA* and *norA* was recorded after the exposure to the combination of Fe16 + AMP, an increased expression of these two genes was also detected with Fe16 and AMP separately. Fe16 alone could increase the expression of *mepA* and *norA* due to the reactive oxygen species that stress the cells and disrupt cellular components. Similar to rifampicin (functionalized iron nanoparticles with anti-tuberculosis drugs), it causes damage to the protein subunits and the chromosome. This could lead to the failure of the efflux pumps [28]. Ampicillin alone also increased the expression of *mepA* and *norA* efflux pumps, as shown for antibiotics similar to β-lactams in other studies [29]. However, the issue with different classes of antibiotics is the residual activity on bacterial targets and the strengthening of the selection of resistance mechanisms [30]. Moreover, the results of the Fe16 and AMP components alone are consistent with the MIC results. As the downregulation of *blaZ* was demonstrated, the association of antibiotics with metals suppresses β-lactamase hydrolase and therefore antibiotics can pass better through the bacterial cell wall [31]. Another indicator of the instability of the defense system of *S. aureus* was a change in the gene expression of the ABC transporters. The overexpression of the ABC transporters under AMP treatment, in our case the *trpABC* gene, suggests rapid adaptation of the reactive immune system and elevation of uptake of iron into the cell which is consistent with the MIC results [32]. On the other hand, the increased expression of *trpABC* and *fhuB* under the influence of Fe16 and Fe16 + AMP and the downregulation of *htsA* in all treatments indicate insufficient uptake of iron by the cell. Inactivation of intramembrane proteolysis of the ABC transporter was found to increase the susceptibility of *S. aureus* to several antimicrobial agents [33]. In another study, the inhibitory effect of zinc on the metal uptake by the ABC transporters at physiological concentrations was demonstrated [34]. However, the number of studies focused on this topic is low and further research is needed. Based on our results, we propose that the application of Fe16 + AMP causes stress in *S. aureus,* facilitates the penetration of AMP through the cell wall, and disrupts the function of efflux pumps and ABC transporters (Fig. 3). This together with insufficient iron intake leads to the bacterial cell death.

In conclusion, Fe16 + AMP is a promising new alternative for treatment of infections caused by *S. aureus* and potentially other pathogenic bacteria. Our results also demonstrate that this combination has no cytotoxic side effects on eukaryotic cells.

## Methods

### Synthesis of Fe16 = [Fe(nphen)_3_](fu)·7H_2_O and preparation of Fe16 + AMP combination

Ferrous fumarate, H_2_fu = fumaric acid and 5-nitro-1,10-phenanthroline (nphen) (Sigma Aldrich, USA) were used for the synthesis of the Fe16 = [Fe(nphen)_3_](fu)·7H_2_O complex. The structure of the Fe16 complex was synthesized on the basis of the already known structure of the individual components mentioned above [35, 36]. Iron fumarate was mixed in 40.0 ml of water with nphen dispersed in the same solvent and stirred for 6.0 h at 40.0 °C. Characterization of Fe16, AMP and Fe16 + AMP was conducted by spectroscopic methods. The iron-antibiotic complex was prepared by mixing Fe16 with AMP in MilliQ water at a concentration of 1.0 mg/ml each and stirred at room temperature for 24 h at 45 rpm.

### Evaluation of synergistic effects between Fe16 and AMP by ζ – potential and infrared spectra (FTIR)

Synergism t between Fe16 and AMP was evaluated by the ζ–potential and infrared spectra (ATR-FTIR) [37, 38]*.* Samples were subjected to analysis of DLS for ζ–potential. The Fourier transform infrared spectrometer equipped with a diamond crystal was used to record infrared spectra of Fe16, AMP and Fe16 + AMP via the attenuated total reflectance method (ATR-FTIR, Vertex 70v, Bruker, Billerica, MA, USA). Detailed methodology is described in the Supplementary file.

### Cultivation of tested bacteria

*Staphylococcus aureus* CCM 4223 (Czech Collection of Microorganisms, Masaryk University, Brno, Czech Republic) was cultured on 5.0% Columbia blood agar (LMS, Czech Republic) at 37.0 °C overnight.

### Evaluation of antimicrobial activity and antimicrobial synergistic effect of Fe16, AMP and Fe16 + AMP by checkerboard assay

Antimicrobial activity and synergistic effect of Fe16, AMP and Fe16 + AMP was tested by the checkerboard assay [39]. Briefly, Fe16 was placed in 96-well microplates diluted twofold in Mueller Hinton broth (Sigma Aldrich, USA) along the vertical rows and AMP was cross-diluted horizontally by twofold serial dilution. Bacterial inoculum of *S. aureus* was added into each well to produce a final concentration of 1–2 × 10^6^ CFU/ml. The plates were incubated at 37 ^◦^C for 24 h. After incubation, the bacterial growth was assessed by observing the color and turbidity of the solution. The tests were carried out in a technical triplicate. The interactions between Fe16 and AMP were evaluated by the fractional inhibitory concentration index (FICI) calculated based on the formula (MIC of A in combination/MIC of A) + (MIC of B in combination/MIC of B) [40].

### Cytotoxic properties of Fe16, AMP and Fe16 + AMP on eukaryotic cell line

Cytotoxic properties of Fe16, AMP, and Fe16 + AMP were evaluated by the spontaneously transformed aneuploidy immortal keratinocyte cell line from the adult human skin (HaCaT). Cell viability was quantified using the MTT assay. Detailed methodology is described in the Supplementary file.

### Cell morphology of *S. aureus* after Fe16, AMP and Fe16 + AMP treatment

Cell morphology of *S. aureus* after Fe16 + AMP treatment and Fe16 and AMP alone was observed by SEM. *Staphylococcus aureus* was mixed with Fe16 + AMP in concentration 0.25 µg/ml (0.125 µg/ml of each Fe16 and AMP), with Fe16 (0.125 µg/ml) and AMP (0.125 µg/ml) alone and cultured at 37.0 °C overnight. After incubation, samples were fixed by glutaraldehyde (1.0%) and incubated for 30 min at room temperature. Samples were then dehydrated using an ascending ethanol series in range 40—100% in several steps. Cell morphology was examined by SEM on the Tescan MAIA 3 equipped with a field emission gun (Tescan Ltd., Brno, Czech Republic). More detailed methods are in the Supplementary file.

### RNA extraction, purification and reverse transcription

For RNA extraction, *S. aureus* was cultured overnight in Luria–Bertani (LB) broth at 37.0 °C and shaking at 120 rpm with and without sub-inhibitory concentrations of 0.25 µg/ml for Fe16, AMP, and Fe16 + AMP. The RNA extraction was performed using TRIzol reagent® (TRIzol Reagent, Invitrogen, Carlsbad, CA) according to the manufacturer instructions. The isolated RNA was purified by ethanol RNA/DNA precipitation and reverse transcription was performed with the transcriptor first strand cDNA synthesis kit for RT-PCR (Roche, Mannheim, Germany) based on the manufacturer instructions using 500.0 ng RNA.

### Quantitative real-time PCR

Quantitative real-time PCR analysis was performed using the qTOWER3 system (Analytik Jena, Jena, Germany) with *rpoB* as the housekeeping gene. Results were visualized as log2 fold change (ΔΔCt) calculations. All primers were designed using the IDT system*.* More detailed methods are in the Supplementary file.

### Statistical evaluation

The unpaired t-test between untreated and treated sample from ΔCt values of biological triplicate was used to determine the impact of treatments on defense system of *S. aureus*. All statistical analysis and graphical visualizations were done using GraphPad Prism 8.0.1. (GraphPad Software, CA, USA).

### Supplementary Information

**Additional file 1: Table S1.** Primers used for Quantitative Real–Time PCR. **Table S2.** Electronic spectral data of aqueous solutions of Fe16. **Table S3.** Expression of the all tested genes of *S. aureus* shown as Fold change after exposure environmental stress of AMP, Fe16 and Fe16+AMP relative to no treated *S. aureus* with use of *rpoB* as housekeeping gene. **Figure S1.** Physico-chemical characterization of the Fe16 complex. **Figure S2.** ζ-potential of Fe16, AMP and Fe16+AMP measured in MiliQ water at pH 8.2.

## Data Availability

Data is available on the department share drive and can be uploaded when requested. The first author may be contacted if someone wants to request the data from this study.

## Abbreviations

AMP: Ampicillin
ATR-FTIR: Attenuated total reflectance-Fourier-transform infrared spectroscopy
DLS: Dynamic light scattering
Fe16: Iron complex
Fe16 + AMP: Combination of iron complex with ampicillin
FIC: Fractional Inhibitory Concentration
MIC: Minimum inhibitory concentration
RPM: Revolutions per minute
