# Supplementary material for: Synergistic antibacterial action of the iron complex and ampicillin against Staphylococcus aureus
Source: BMC Microbiol. 2023 Oct 6;23:288. doi: 10.1186/s12866-023-03034-1 (PMC10559456; doi:10.1186/s12866-023-03034-1)
Supplement: Supplementary file 1 — Additional file 1: Table S1. Primers used for Quantitative Real–Time PCR. Table S2. Electronic spectral data of aqueous solutions of Fe16. Table S3. Expression of the all tested genes of S. aureus shown as Fold change after exposure environmental stress of AMP, Fe16 and Fe16+AMP relative to no treated S. aureus with use of rpoB as housekeeping gene. Figure S1. Physico-chemical characterization of the Fe16 complex. Figure S2. ζ-potential of Fe16, AMP and Fe16+AMP measured in MiliQ water at pH 8.2. [file 12866_2023_3034_MOESM1_ESM.docx]

**Supplementary data**

**Table S1.** Primers used for Quantitative Real–Time PCR

| Primer name | Sequence | Amplicon size | References |  |
| --- | --- | --- | --- | --- |
| *blaZ-F*  *blaZ-R* | ACAACTGTAATATCGGAGGGTTTAT  TCTTTGGCATGTGAACTGTTTG | 103 | This study | |
| *norA-F*  *norA-R* | ATGAATAAACAGATTTTTGT  CTACATATTTTGTTCTTTCA | 167 | ([1](#_ENREF_1)) | |
| *Mep-F*  *Mep-R* | TGTGAAGGTGTTGTACCACTAA  ACAAAGATAACGCCGATAGACA | 104 | ([2](#_ENREF_2)) | |
| *trpABC-F*  *trpABC-R* | TGTAGCAGTAGCGGGTTGTG  TTAGTGCTGCCATCATCTGC | 192 | ([3](#_ENREF_3)) | |
| *fhuB-F*  *fhuB-R* | TAGCAGCAGTAATTGTAGGTATGG  CTAAAGCAAATGAAGCACCTGAA | 123 | ([1](#_ENREF_1)) | |
| *htsA-F*  *htsA-R* | CAGAAGAAATTAAGCCACGAGAT  ACAAGAAGATGCAACATGGAAA | 104 | ([4](#_ENREF_4)) | |
| *rpoB-F*  *rpoB-R* | GCATTAGGACCTGGTGGTTTA  TTTGGTCCCTCAGGTGTTTC | 110 | This study | |

**Table S2.** Electronic spectral data of aqueous solutions of Fe16.

| *λ (nm)* | *ε (L · mol^–1^ · cm^–1^)* | *Assignment* |
| --- | --- | --- |
| *237* | *5.6 × 10^4^* | *π → π** |
| *271* | *7.7 × 10^4^* | *π → π** |
| *320* | *1.7 × 10^4^* | *π → π** |
| *478* | *1.0 × 10^4^* | *MLCT* |
| *510* | *1.1 × 10^4^* | *MLCT* |

**Table S3**. Expression of the all tested genes of *S. aureus* shown as Fold change after exposure environmental stress of AMP, Fe16 and Fe16+AMP relative to no treated *S. aureus* with use of *rpoB* as housekeeping gene.

| **Fold change** | | | | |
| --- | --- | --- | --- | --- |
|  | **Control** | **AMP** | **Fe16** | **Fe16+AMP** |
| *trpABC* | 1 | 14.38 | 3.18 | 3.18 |
| *fhuB* | 1 | 1.23 | 2.73 | 2.50 |
| *htsA* | 1 | 0.77 | 0.60 | 0.63 |
| *mepA* | 1 | 3.43 | 6.23 | 9.33 |
| *norA* | 1 | 2.80 | 1.93 | 3.10 |
| *blaZ* | 1 | 0.68 | 0.50 | 0.52 |

**
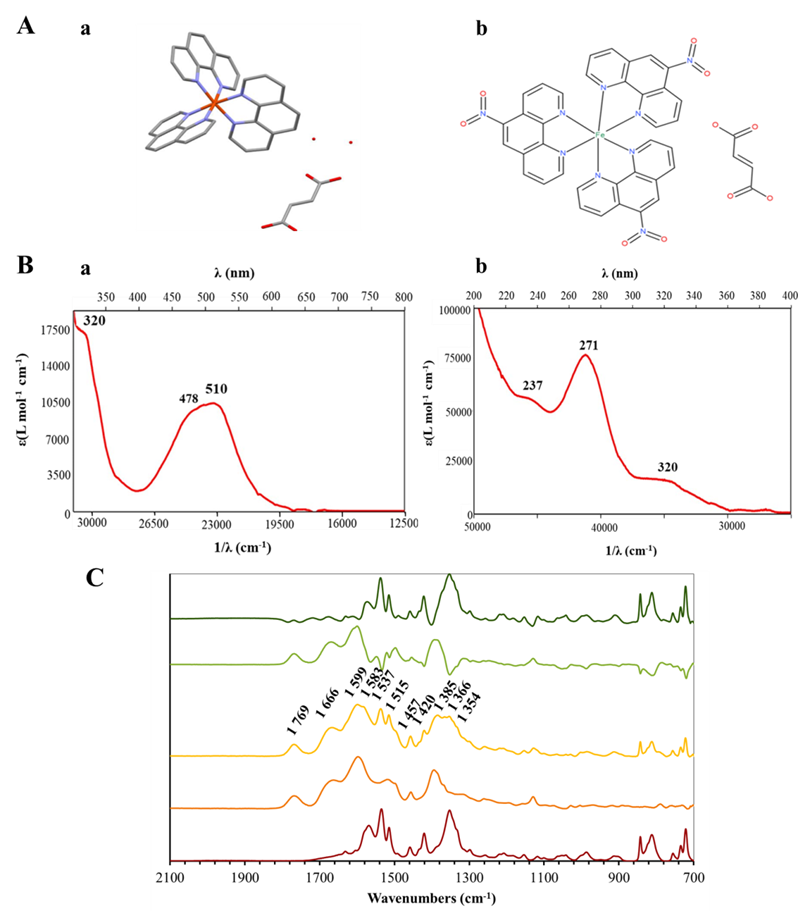
**

***Figure S1.*** *Physico-chemical characterization of the Fe16 complex.* ***A.*** *molecular structure of [Fe(phen)3)](fu)·2H_2_O, hydrogen atoms are omitted for clarity. Iron (red) is coordinated by six nitrogens (blue), oxygen atoms of fumarate and oxygen atoms of water molecules are in red (****a****). Proposed structure of Fe16 with omitted water molecules (****b****).* ***B.*** *Electronic absorption spectra of aqueous solutions of Fe16 at concentration c = 0.1 mmol/L (****a****) and (****b****) 20.0 µmol/L.* ***C.*** *ATR-FTIR analysis of Fe16 recorded spectrum (yellow line), Fe16 subtracted spectrum (orange line), AMP recorded spectrum (light green line), AMP subtracted spectrum (dark green line), and Fe16+AMP recorded spectrum (black line). Spectra are shown in cropped spectral region with significant vibration bands of 2000 - 700 cm-1. All spectra are recorded in absorbance mode and are depicted with offset Y values.*

**
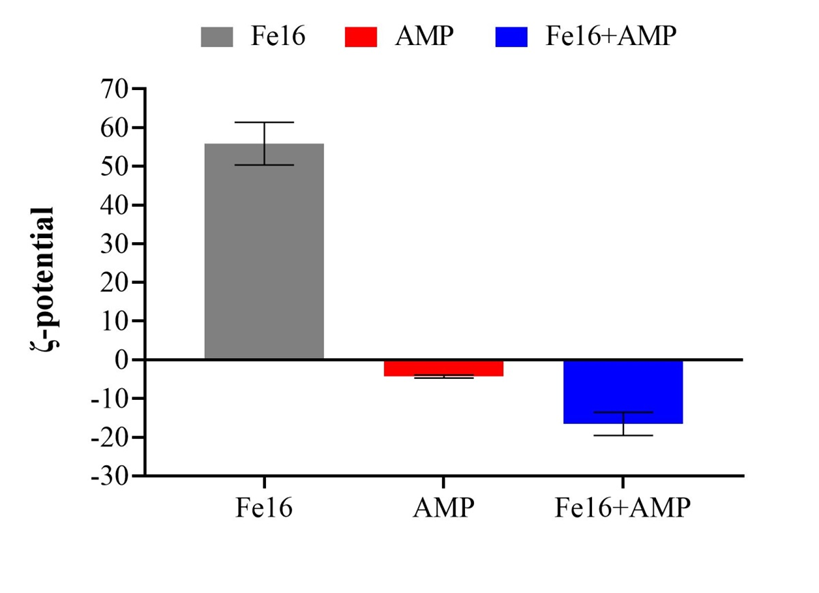
**

***Figure S2.*** ζ-*potential of Fe16, AMP and Fe16+AMP measured in MiliQ water at pH 8.2*

**Methodology**

*Synthesis of Fe16* = [Fe(nphen)_3_](fu) ·7H_2_O

Iron(II) fumarate, H_2_fu = fumaric acid and 5-nitro-1,10-phenanthroline (nphen) were obtained from Sigma Aldrich. Iron fumarate (0.17 g; 1.0 mmol) was mixed in 40 ml of water with nphen (0.68 g; 3.0 mmol) dispersed in the same solvent. The mixture was stirred for 6 h at 40 ˚C. The dark red solution was filtered and left for crystallization. Dark red crystals obtained after a week were collected on a frit and dried at room temperature. Yield: 0.64 g.Anal. Calc.: C, 49.3; H, 4.0; N, 12.9. Found: C, 49.1; H, 3.8; N, 12.5%. IR (ATR, cm^–1^): 1564 m, 1530 s, 1512 s, 1456 m, 1418 m, 1343 s, 1297 sh, 1152 m, 982 m, 808 m, 718 m, 654 w. UV-VIS (*λ*, nm): 237, 271, 320, 478, 510.

*Characterization of Fe16, AMP, and Fe16+AMP by Spectroscopy Methods*

Infrared (IR) spectra were recorded on an FTIR Jasco FT/IR-4700 (Jasco, Eaton, MD, USA). Absorption spectra of aqueous solutions were measured on a Cintra 3030 spectrometer (GBC Scientific Instruments, IL, USA) in 1 cm quartz cuvettes at room temperature. The spectra were measured immediately after dissolution. A baseline was measured by use of a sample and reference cells filled with pure solvent before measurements. Solid state absorption spectra were measured as a mull of crystalline powder in nujol at room temperature.

*Evaluation of Synergistic Effects between Fe16 and AMP by* ζ*–Potential and Infrared Spectra (ATR-FTIR)*

Fe16 and AMP were diluted in MiliQ water to concentration 1 mg/mL, Fe16+AMP was prepared as described above. The 1 mL of diluted samples were transferred in to disposable cuvettes ZEN0040 (BRAND GMBH + CO KG,Wertheim, Germany), enclosed with stoppers and placed in detector angle of 173°, refractive index of 1.33 at 25 °C of ZetaSizer Nano ZS (Malvern). Fe16 and AMP were diluted in MiliQ water to concentration 1 mg/mL, Fe16+AMP was prepared as described above. The 1 mL of diluted samples were transferred in to disposable cuvettes ZEN0040 (BRAND GMBH + CO KG,Wertheim, Germany), enclosed with stoppers and placed in detector angle of 173°, refractive index of 1.33 at 25 °C of ZetaSizer Nano ZS (Malvern). Samples were subjected to analysis of DLS to determine the ζ–potential and verification of synergy combination Fe16+AMP compared to Fe16 and AMP alone. The equilibration time was 120 sec, and the measurements were performed in hexaplicates.

Fourier transform infrared spectrometer equipped with a diamond crystal was used to record infrared spectra of Fe16, AMP and Fe16+AMP via the attenuated total reflectance method (ATR-FTIR, Vertex 70v, Bruker, Billerica, MA, U.S.A.). Samples were measured in powder form, Fe16+AMP powder was collected by freeze drying. The spectra were acquired in the range of 4500 to 700 cm^−1^. To obtain one spectrum, 128 scans of spectra with a resolution of 2 cm^−1^ were averaged. Spectrum processing (standardization, subtraction) was performed in the Origin 2020b program (Origin Lab, Northampton, MA, U.S.A).

*Evaluation of Antimicrobial Activity and Synergistic effect of Fe16, AMP and Fe16+AMP by Checkerboard analysis*

The *Staphylococcus aureus* CCM 4223 was diluted in Mueller Hinton broth (Sigma Aldrich, USA) to 0.5 Mc Farland units and then diluted 100 × to reach cell density 1–2 × 10^6^ CFU/mL. 100 μL of prepared bacterial inoculum was placed in 96-well microplates and 50 μL of Fe16 and AMP in MH was added. Fe16 was diluted twofold in MH along the vertical rows and AMP was cross-diluted horizontally by twofold serial dilution. Final volume of each well was 200 μL and final concentration range of Fe16 was 125-2 μg/ml and AMP 63-0.063 μg/ml The plates were incubated at 37 ^◦^C for 24 h. After incubating, the bacterial growth was assessed by observing the color and turbidity of the solution. The measurements were performed in technical triplicates ([5](#_ENREF_5)).

The interactions between antimicrobial agents (Fe16 and AMP) were evaluated by the FIC index, which was calculated according to the formula (MIC of A in combination/MIC of A) + (MIC of B in combination/MIC of B). The interaction was defined as synergistic if the FIC index was ≤ 0.5, additive if it was > 0.5 but ≤ 1, indifferent if it was 2 and antagonistic if it was > 2 ([6](#_ENREF_6)).

*Cytotoxic Properties of Fe16, AMP and Fe16+AMP on Eukaryotic Cell Line*

Spontaneously transformed aneuploidy immortal keratinocyte cell line from adult human skin (HaCaT) was cultured in RPMI–1640DMEM medium with 10 % fetal bovine serum, supplemented with penicillin (100 U/mL) and streptomycin (0.1 mg/mL). Cells were harvested, washed four times with PBS (pH 7.4) and counted using Countess IIFL Automated Cell Counter (Life Technologies, Carlsbad, CA, USA). Cell viability was estimated using the MTT assay. The suspension of 5,000 cells in 50 µL medium was added to each well of microtiter plates (E-plates 96), followed by incubation for 24 h at 37 °C with 5 % CO_2_ to ensure the cell growth. The treatment was initiated after the cells reached ~ 60 - 80 % confluence. 50 µl of medium containing Fe16, AMP and Fe16+AMP in concentrations of 500 - 0.5 µg/mL was employed. Treated cells were incubated for 24 h. Further, 10 µL of MTT (5 mg/mL in PBS [3-(4,5-dimethylthiazol-2-yl)-2,5-diphenyltetrazolium bromide]) was added to the cells and the mixture was incubated at 37 °C for 4 h. MTT-containing medium was replaced by 100 µL of 99.9 % dimethyl sulfoxide to dissolve MTT-formazan crystals and, after 5 min incubation, absorbance of the samples was measured at 570 nm (VersaMax microplate reader, Molecular Devices, Sunnyvale, CA, USA).

*Cell Morphology of S. aureus After Fe16+AMP Treatment*

Staphylococcus aureus was diluted in 2 × Mueller Hinton broth to 0.5 Mc Farland units and mixed with Fe16+AMP in concentration 0.25 µg/mL. These samples were cultured at 37 °C overnight. After incubation, the samples were centrifuged (1000 × g, 5 min). 1 ml of PBS was added to the pellet and incubated at 37 °C/45 min/600 RPM. Then the samples were centrifuged (3000 × g, 2 min) and washed three times with PBS. Glutaraldehyde (1%) was added to the pellet and incubated 30 min in the dark at room temperature. After incubation, the samples were washed three times by MilliQ water, when 1 mL of MilliQ water was added, incubated 10 min and centrifuged (3000 × g, 2 min). The washed samples were dehydrated using an ascending ethanol series in range 40 - 100 % in several steps. Each time, the appropriate percentage of ethanol was added to the samples, incubated 5 min, and centrifuged (3000 × g, 2 min). Samples with 100 % ethanol were incubated for 5 and 15 min, washed with 2 × 100 % ethanol and centrifuged (3000 × g, 2 min). The morphology was examined by scanning electron microscopy on a Tescan MAIA 3 equipped with a field emission gun (Tescan Ltd., Brno, Czech Republic). Best images were obtained using the In-Beam SE detector at working distance was approximately 3.00 mm and at 2 kV acceleration voltages. 768 × 858 pixels images were obtained at 22,100-fold magnification covering sample area of 9.392 µm2. Full frame capture was performed in UHresolution mode and accumulation of image with image shift correction enabled, and it took about 0.5 min with the ∼1 µs/pixel dwell time. Spot size was set at 4.14 nm.

*RNA Extraction, Purification and Reverse Transcription*

*Staphylococcus aureus* was grown overnight in Luria-Bertani (LB) broth at 37 °C and 120 RPM with or without sub-inhibitory concentrations (0.25 µg/mL) of Fe16, AMP, and Fe16+AMP. RNA extractions of *S. aureus* were performed using TRIzol Reagent® (TRIzol Reagent, Invitrogen, Carlsbad, CA) according to the manufacturer’s instructions. The 5 µg of total RNA was DNase treated using TURBO™ DNase freeTM Kit (Ambion®, Life Technologies Europe BV) to remove remaining genomic DNA and complete removal of contaminating DNA was confirmed by PCR. The isolated RNA was purified by ethanol RNA/DNA precipitation protocol according to the manufacturer's instructions. Reverse transcription was performed with the Transcriptor first strand cDNA synthesis kit for RT-PCR (Roche, Mannheim, Germany) according to the manufacturer's instructions using 500 ng RNA. Prepared cDNA was diluted in UltraPure™Dnase/Rnase-Free Destiled Water to a total volume of 200 µL.

*Quantitative Real-Time PCR*

Analysis was performed using qTOWER3 system (Analytik Jena, Jena, Germany). 10 μL of diluted reverse-transcribed cDNA was subjected to PCR amplification in a 20 μL final volume containing Luna® Universal qPCR Master Mix (New England Biolabs) following the manufacturer’s instructions with the 5 pmol of each of the specific primers diluted in UltraPureTM DNase/RNase-Free Distilled Water for each reaction. The amplification conditions were: 1 cycle at 98 °C for 3 min and 35 cycles at 95 °C for 15 sec and 60 °C for 40 sec. To confirm the specificity of the PCR, melting curve analysis was performed. To calculate relative expression, *rpoB* was used as a reference gene. The results were visualized as Log2 fold change (ΔΔCt) calculations. All used primers were designed using the IDT system.

**References**

1. Deng X, Sun F, Ji Q, Liang H, Missiakas D, Lan L, et al. Expression of multidrug resistance efflux pump gene norA is iron responsive in Staphylococcus aureus. J Bacteriol. 2012;194(7):1753-62.

2. Opperman TJ, Williams JD, Houseweart C, Panchal RG, Bavari S, Peet NP, et al. Efflux-mediated bis-indole resistance in Staphylococcus aureus reveals differential substrate specificities for MepA and MepR. Bioorganic & Medicinal Chemistry. 2010 2010/03/15/;18(6):2123-30.

3. Allard M, Moisan H, Brouillette É, Gervais AL, Jacques M, Lacasse P, et al. Transcriptional modulation of some Staphylococcus aureus iron-regulated genes during growth in vitro and in a tissue cage model in vivo. Microbes and Infection. 2006 2006/06/01/;8(7):1679-90.

4. Beasley FC, Vinés ED, Grigg JC, Zheng Q, Liu S, Lajoie GA, et al. Characterization of staphyloferrin A biosynthetic and transport mutants in Staphylococcus aureus. Molecular microbiology. 2009;72(4):947-63.

5. Khunbutsri D, Naimon N, Satchasataporn K, Inthong N, Kaewmongkol S, Sutjarit S, et al. Antibacterial Activity of Solanum torvum Leaf Extract and Its Synergistic Effect with Oxacillin against Methicillin-Resistant Staphyloccoci Isolated from Dogs. Antibiotics. 2022;11:302.

6. Mgbeahuruike EE, Stålnacke M, Vuorela H, Holm Y. Antimicrobial and Synergistic Effects of Commercial Piperine and Piperlongumine in Combination with Conventional Antimicrobials. Antibiotics. 2019;8(2):55.
